# Racial and Ethnic Disparities in Pain Management of Children With Limb Fractures or Suspected Appendicitis: A Retrospective Cross-Sectional Study

**DOI:** 10.3389/fped.2021.652854

**Published:** 2021-08-03

**Authors:** Romain Guedj, Maddalena Marini, Joe Kossowsky, Charles B. Berde, Amir A. Kimia, Eric W. Fleegler

**Affiliations:** ^1^Department of Anesthesiology, Critical Care and Pain Medicine, Boston Children's Hospital, Boston, MA, United States; ^2^Department of Pediatric Emergency Medicine, Trousseau Hospital, Assistance Publique des Hôpitaux de Paris, Sorbonne Université, Paris, France; ^3^Obstetrical, Perinatal, and Pediatric Epidemiology Research Team, Epidemiology and Statistics Research Center, Université de Paris, INSERM, Paris, France; ^4^Istituto Italiano di Tecnologia, Center for Translational Neurophysiology, Ferrara, Italy; ^5^Department of Anaesthesia, Harvard Medical School, Boston, MA, United States; ^6^Division of Emergency Medicine, Boston Children's Hospital, Boston, MA, United States; ^7^Departments of Pediatrics and Emergency Medicine, Harvard Medical School, Boston, MA, United States

**Keywords:** pain management, children, racial and ethnic differences, equitable care, pediatric emergency, disparities, appendicitis, fracture

## Abstract

**Objective:** To evaluate whether racial/ethnical differences in analgesia administration existed in two different cohorts of children with painful conditions: children with either limb fracture or suspected appendicitis.

**Methods:** Retrospective cross-sectional analysis of children visiting a pediatric emergency department (Boston Children Hospital) for limb fracture or suspected appendicitis from 2011 to 2015. We computed the proportion of children that received any analgesic treatment and any opioid analgesia. We performed multivariable logistic regressions to investigate race/ethnicity differences in analgesic and opioid administration, after adjusting for pain score, demographics and visit covariates.

**Results:** Among the 8,347 children with a limb fracture and the 4,780 with suspected appendicitis, 65.0 and 60.9% received any analgesic treatment, and 35.9 and 33.4% an opioid analgesia, respectively. Compared to White non-Hispanic Children, Black non-Hispanic children and Hispanic children were less likely to receive opioid analgesia in both the limb fracture cohort [Black: aOR = 0.61 (95% CI, 0.50–0.75); Hispanic aOR = 0.66 (95% CI, 0.55–0.80)] and in the suspected appendicitis cohort [Black: aOR = 0.75 (95% CI, 0.58–0.96); Hispanic aOR = 0.78 (95% CI, 0.63–0.96)]. In the limb fracture cohort, Black non-Hispanic children and Hispanic children were more likely to receive any analgesic treatment (non-opioid or opioid) than White non-Hispanic children [Black: aOR = 1.63 (95% CI, 1.33–2.01); Hispanic aOR = 1.43 (95% CI, 1.19–1.72)].

**Conclusion:** Racial and ethnic disparities exist in the pain management of two different painful conditions, which suggests true inequities in health care delivery. To provide equitable analgesic care, emergency departments should monitor variation in analgesic management and develop appropriate universal interventions.

## Introduction

Racial and ethnic disparities in both medical management and medical outcomes are well-documented in the adult medical literature (1, 2). A recent systematic review found that Black non-Hispanic (NH) and Hispanic adults were less likely than White NH adults to receive analgesia for acute pain in the emergency department (ED) (2). Research on health inequalities in pain assessment and treatment among children in pediatric emergency departments (PED) have shown mixed results (3). Studies using data from national surveys or multicentric studies have consistently shown racial and ethnic differences in pain management in the ED overall (4, 5) but heterogeneous results for appendicitis (6, 7) and limb fractures (8, 9). Other studies, mainly retrospective and monocentric, find no racial differences in analgesic management of bone fractures or appendicitis(10–13).

Abdominal and extremity pain represent between 35 and 40% of pain-related US ED visits (14). Opioid management in the ED is used in about 15% of abdominal pain cases and in about 40% of PED visits for fracture pain (4). Thus, abdominal pain/appendicitis and limb fractures have provided a convenient paradigm to evaluate racial/ethnic differences in analgesia management in PED (6–12, 15). However, the majority of these pediatric studies look either at the use of analgesia in single conditions or the use or prescription of opioids across “potentially painful conditions” without data about the presence or severity of pain (5), and therefore miss an opportunity to look at the pervasiveness of potential pain management disparities across multiple specific painful conditions in a given setting.

We aimed to evaluate during the same time period whether racial/ethnic disparities in analgesia administration existed in two different cohorts of children presenting with painful conditions: children with either limb fractures or suspected appendicitis. We hypothesized that Black NH children and Hispanic children would consistently receive pain management at rates lower than White NH children.

## Methods

### Study Design and Setting

We performed a cross-sectional retrospective analysis of two cohorts of patients presenting to the PED of a single large urban pediatric tertiary center (Boston Children Hospital, Boston, USA): visits of patients with a limb fracture and visits of patients with abdominal pain with suspected appendicitis. We determined and compared the proportion of children with any analgesic treatment, non-opioid and opioid treatment according to the race/ethnicity of the patients. The study was approved by the Boston Children's Hospital Institutional Review Board.

### Selection of Participants

The limb fracture cohort included visits of children from 2 to 18 years of age, with a diagnosis of limb fracture between January 2011 and September 2015. We determined this age threshold based on the literature suggesting that the use of opioids is lower in infants (4). The exclusion criteria included patients with a complex chronic condition (16) and patients with an associated head, thoracic, abdominal or spine injury. For this cohort, visits were identified by querying the following International Classification of Diseases-9 (ICD-9) discharge diagnoses codes (primary and others): 812 (fracture of humerus), 813 (fracture of radius and ulna), 820 (fracture of the neck of femur), 821 (fracture of other and unspecified parts of femur), 823 (fracture of tibia or fibula), and 824 (fracture of ankle).

The suspected appendicitis cohort included children aged 5–18 years, presenting with abdominal pain concerning for appendicitis between January 2011 and December 2015. Children under the age of 5 years have a very low rate of appendicitis (1.1 per 10,000) and are less likely than older children to be evaluated for appendicitis (17). Children with complex chronic conditions were excluded.

In order to identify this cohort, we created a clinician-supervised artificial intelligence (AI) methodology (combining manual review and machine learning techniques). In brief, we defined a visit with an evaluation for appendicitis as a visit with both (1) a complaint of abdominal pain and a suspicion of appendicitis noted by the ED physician in the clinical note and (2) an abdominal ultrasound, CT or MRI scan performed to rule in or out appendicitis. In order to train an AI model, 1 year of clinical ED notes were manually reviewed using a computer-assisted key word screening tool using regular-expression matching in order to create a set of labeled clinical notes (18). This technique has been used in prior studies (19, 20). Next the notes identified with regular expression matching were fed into a document classifier using n-gram methods. In short, a document classifier uses the training set containing documents of interest and those that are not and creates a model that searches new document sets for documents similar to the documents of interest. Search engines like GOOGLE and YAHOO are examples of document classifiers. This method, unlike key word search, provides metrics to the search (accuracy, sensitivity, etc.) We trained a document classifier with a portion of our data and validated it with a separate manually reviewed validation set. Next, we combined the AI capability with human expertise: We used the model to screen every clinical note from 2011 to 2015 (281,970 in total) in order to classify the documents to include and exclude. Medical records with very high and very low likelihood of inclusion were automatically classified. Medical records with an intermediate probability were manually reviewed. We performed quality measurements by reviewing 10% of the included documents and 10% of the excluded documents and found 4.6% false positive rate and 2.7% false negative rate (i.e., missing cases).

### Measurements

All patient and visit variables were retrieved *via* structured data forms from the ED electronic medical records (EMR).

During ED registration, families were asked to provide the race and ethnicity of their child; families could choose to decline to answer. We classified race/ethnicity as “White Non-Hispanic,” “Black non-Hispanic,” “Hispanic,” and “other” (including Asian, American Indian, Alaska Native, Native Hawaiian and other Pacific Islander, and self-labeled “other”; these categories were aggregated due to low numbers in the ED.)

Pain measurements were performed by a nurse in triage and later by nurses or clinical assistants while in the emergency department. To measure pain in children ages 2–4, the FLACC Scale (Face, Legs, Activity, Cry Consolability) was utilized (21). The scale is scored in a range of 0–10 with 0 representing no pain. Among children age 5–7, the Wong-Baker FACES Pain Rating Scale was used (0–10 in 2-point increments) and in children ages 8–18 the Numeric Rating Scale (0–10) was utilized per emergency department protocols (22).

The covariates used in both cohorts included sex, age, median income by zip code, insurance type (public, private, self-pay), initial pain score and triage acuity level. We chose these covariates because they were *a priori* considered to have a potential relationship with analgesic and opioid administration based on prior literature (6, 9, 12). Specific covariates for the limb fracture cohort included location of the fracture (upper or lower limb) as well as receipt of sedation in the ED. The specific covariate for the suspected appendicitis cohort included surgery or interventional radiology drainage for appendicitis. Initial pain score included the first pain score evaluation in the ED. Pain scores were recorded using a 10-point scale and we categorized initial pain score as none (0), mild (1–3), moderate (4–6), or severe (7–10). Triage acuity level utilized the 5-level Emergency Severity Index (ESI) (23). We collapsed the triage acuity level into three categories: 1–2 (highest acuity), 3, and 4–5 (lowest acuity) ESI level.

### Outcomes

All of the administrations of analgesic drugs by any route, including acetaminophen, ibuprofen, ketorolac, morphine, fentanyl and hydromorphone, during the ED visits were retrieved then categorized as opioid or non-opioid treatment. We defined (1) any analgesic treatment, when at least one non-opioid or opioid treatment was administered; (2) only non-opioid analgesia, when analgesic treatments did not include any opioid treatment; and (3) opioid analgesia, when analgesic treatments included at least one opioid treatment.

Our primary outcome was the administration of any opioid analgesia during the ED stay with secondary outcomes of any analgesic treatment and only non-opioid analgesia.

### Analyses

Both cohorts were analyzed independently with the same analytic steps. Data were reported as means for continuous variables and proportions for categorical variables. We performed bivariate analyses to study the association of race/ethnicity on any analgesic treatment, only non-opioid analgesia and opioid analgesia overall as well as stratified by pain score. We also performed bivariate analyses to study the association of the covariates with opioid analgesia and any analgesic treatment by using chi square for categorial variables and *t*-test for continuous variables. Finally, we performed multivariable logistic regressions to adjust for confounding by the covariates listed above. Results from the multivariable model included adjusted odds ratios (aOR). Patients with missing data were excluded from the multivariate analyses. The analyses were performed using STATA Version 14. We used the Strengthening the Reporting of Observational Studies in Epidemiology (STROBE) methodology to report the results (24).

## Results

Flow charts for both cohorts are shown in Appendix 1.

### Limb Fracture Cohort

There were 8,357 visits for limb fractures during the study period. Data about race/ethnicity were available for 7,207 (86.2%) visits. The distribution included 69.2% White NH, 10.7% Black NH, 13.8% Hispanic and 6.2% other. The initial pain score was moderate or severe for almost 60% of those visits (see Table 1). Black NH and Hispanic children were more likely to have a severe initial pain score than White NH children (Black NH = 38.7% and Hispanic = 33.5% vs. White NH = 23.6%, *p* < 0.001).

**Table 1:** Visit characteristics of children with limb fracture (*N* = 8,357)^a^.

**Characteristics**	***N*** **(%)**	**Missing**
**Female**	3,356 (40.2)	0 (0)
**Age (years), mean (SD)**	9.3 (4.2)	0 (0)
**Race/ethnicity**		1,150 (13.8)
White, non-Hispanic	4,989 (69.2)	
Black, non-Hispanic	772 (10.7)	
Hispanic	996 (13.8)	
Other	450 (6.2)	
**Insurance type**		0 (0)
Private	5,756 (68.9)	
Public	2,542 (30.4)	
None	59 (0.7)	
**Median household income by zip code ($), mean (SD)** ^**b**^	82,133 (30,773)	53 (0.6)
**ESI Triage score**		60 (0.7)
1–2	695 (8.4)	
3	6,893 (83.1)	
4–5	709 (8.5)	
**Pain score**		83 (1)
None (0)	1,686 (20.4)	
Mild (1–3)	1,727 (20.9)	
Moderate (4–6)	2,726 (33.0)	
Severe (7–10)	2,135 (25.8)	
**Fracture location**		0 (0)
Upper limb	6,151 (73.6)	
**Sedation in the ED**	1,846 (22.1)	0 (0)

a *Each visit for an acute limb fracture was considered independently; if a given patient had more than one fracture during the time period, the receipt of medications would be considered separately*.

b *Each patient has a median income for zip code assigned. The mean of these income values is shown here*.

#### Analgesia Modalities

Overall, 65.0% of limb fracture visits received at least one analgesic (opioid or non-opioid). 35.9% of visits for limb fractures received an opioid.

Table 2 shows the distribution of analgesia receipt by race/ethnicity. In bivariate analyses (Appendix 2), Black NH children were more likely to receive any analgesic treatment [OR = 1.69 (95% CI, 1.42–2.01)], more likely to receive only non-opioid analgesia [OR = 2.59 (95% CI 2.22–3.03)] and less likely to receive an opioid analgesia [OR = 0.63 (95% CI 0.54–0.75)] than White NH children. Hispanic children were more likely to receive any analgesic treatment [OR = 1.36 (95% CI, 1.17–1.58)], more likely to receive only non-opioid analgesia [OR = 2.20 (95% CI, 1.91–2.53)] and less likely to receive an opioid analgesia [OR = 0.63 (95% CI, 0.54–0.73)] than White NH children. The “other” racial group children were more likely to receive only non-opioid analgesia [OR = 1.48 (95% CI 1.20–1.82)] and less likely to receive an opioid analgesia than White NH children [OR = 0.73 (95% CI, 0.60–0.90)]. The utilization of pain medications by initial pain score and by race/ethnicity are shown in Appendix 3.

**Table 2:** Unadjusted analgesia modalities by race/ethnicity for limb fracture and suspected appendicitis visits.

	**Number and proportion of children by race**						
**Race/ethnicity**	**No analgesia**		**Only non-opioid analgesia**		**Opioid analgesia**		***p***
	***n***	**% (95%CI)**	***n***	**% (95%CI)**	***n***	**% (95%CI)**	
**Limb fracture cohort**							
• White non-Hispanic (*N* = 4,989)	1,838	36.8 (25.5–38.1)	1,224	24.5 (23.4–25.7)	1,927	38.6 (37.3–40.0)	
• Black non-Hispanic (*N* = 772)	198	25.7 (22.7–28.9)	353	45.7 (42.2–49.3)	221	28.6 (25.5–31.9)	
• Hispanic (*N* = 996)	299	30.0 (27.2–32.9)	415	41.7 (38.6–44.8)	282	28.3 (25.6–31.2)	
• Others (*N* = 450)	162	36.0 (31.7–40.6)	146	32.4 (28.3–36.9)	142	31.6 (27.4–36.0)	<0.001
**Suspected appendicitis cohort**							
• White non-Hispanic (*N* = 2,599)	1,058	40.7 (38.8–42.6)	635	24.4 (22.8–26.1)	906	34.9 (33.0–36.7)	
• Black non-Hispanic (*N* = 486)	170	35.0 (30.9–39.3)	164	33.7 (29.7–38.1)	152	31.3 (27.3–35.6)	
• Hispanic (*N* = 900)	292	32.4 (29.5–35.6)	306	34.0 (31.0–37.2)	302	33.6 (30.5–36.7)	
• Others (*N* = 235)	106	45.1 (38.8–51.6)	70	29.8 (24.2–36.0)	59	25.1 (20.0–31.1)	<0.001

#### Analgesic Treatment and Opioid Analgesia: Multivariate Analyses

In the multivariate analyses (Table 3), Black NH children were more likely to receive any analgesic treatment than White NH children [aOR = 1.63 (95%, 1.33–2.01)] but less likely to receive an opioid analgesia [aOR = 0.61 (95% CI, 0.50–0.75)]. Hispanic children were more likely to receive any analgesic treatment than White NH children [aOR = 1.43 (95% CI, 1.19–1.72)] but less likely to receive an opioid analgesia [aOR = 0.66 (95% CI, 0.55–0.80)].

**Table 3:** Factors associated with the administration of any analgesic treatment and opioid analgesia for children visiting the emergency department with a limb fracture.

	**Any analgesic treatment**	**Opioid analgesia**
	**OR (95%-CI)**	**OR (95%-CI)**
**Race, ethnicity %**		
White non-Hispanic	1	1
Black non-Hispanic	**1.63 (1.33–2.01)**	**0.61 (0.50–0.75)**
Hispanic	**1.43 (1.19–1.72)**	**0.66 (0.55–0.80)**
Other	1.19 (0.95–1.49)	0.87 (0.69–1.09)
**Sex**		
Male	1	1
Female	0.98 (0.88–1.10)	**0.84 (0.75–0.94)**
**Age, years**	**0.95 (0.94–0.96)**	0.99 (0.98–1.01)
**Insurance status**		
Public	1	1
Private	**1.24 (1.08–1.42)**	1.09 (0.95–1.25)
No insurance	1.14 (0.58–2.24)	1.01 (0.52–1.94)
**Median household income by ZIP code in $**	1.00 (1.00–1.00)	1.00 (1.00–1.00)
**Triage score**		
1–2	1	1
3	**0.24 (0.19–0.29)**	**0.24 (0.19–0.29)**
4–5	**0.03 (0.02–0.05)**	**0.03 (0.02–0.05)**
**Pain score group, %**		
None (0)	1	1
Mild (1–3)	**1.77 (1.52–2.07)**	**1.46 (1.21–1.75)**
Moderate (4–6)	**4.34 (3.74–5.04)**	**2.22 (1.88–2.63)**
Severe (7–10)	**13.49 (11.10–16.39)**	**3.99 (3.33–4.79)**
**Fracture Location**		
Lower limb	1	1
Upper limb	**0.80 (0.70–0.91)**	**0.65 (0.58–0.74)**
**Sedation**		
No	1	1
Yes	1.03 (0.90–1.18)	**2.26 (1.99–2.56)**

*Multivariate analyses. Bold values indicate statistically significant*.

### Suspected Appendicitis Cohort

There were 4,780 visits for suspected appendicitis during the study period. Data about race/ethnicity were available for 4,220 (88.3%) visits. The distribution included 61.9% White NH, 11.2% Black NH, 21.3% Hispanic and 5.6% other. The initial pain score was moderate or severe for 76% of the suspected appendicitis cohort (see Table 4). Black NH and Hispanic children were more likely to have a severe initial pain score than White NH children (Black NH = 49.8% and Hispanic = 52.3% vs. White NH = 35.1%, *p* < 0.001).

**Table 4:** Visit characteristics of children with suspected appendicitis (*N* = 4,780)^a^.

**Characteristics**	***N*** **(%)**	**Missing**
**Female**	2,703 (56.6)	(0)
**Age (years), mean (SD)**	12.0 (3.9)	(0)
**Race/ethnicity**		560 (11.7)
White, non-Hispanic	2,599 (61.6)	
Black, non-Hispanic	486 (11.5)	
Hispanic	900 (21.3)	
Other	235 (5.6)	
**Insurance type**		11 (0.2)
Private	3,134 (65.7)	
Public	1,603 (33.6)	
None	32 (0.7)	
**Median household income by zip code ($), mean (SD)** ^**b**^	78,280 (30,021)	43 (0.9)
**Triage score**		22 (0.5)
1–2	454 (9.5)	
3	4,170 (87.7)	
4–5	134 (2.8)	
**Pain score**		199 (4.2)
None (0)	546 (11.9)	
Mild (1–3)	553 (12.1)	
Moderate (4–6)	1,682 (36.7)	
Severe (7–10)	1,899 (39.3)	
**Surgery for appendicitis**	1,411 (29.5)	0 (0)

a *Each visit for an suspected appendicitis was considered independently; if a given patient had more than one visit for suspected appendicitis in the time period the receipt of medications would be considered separately*.

b *Each patient has a median income for zip code assigned. The mean of these income values is shown here*.

#### Analgesia Modalites

Overall, 60.9% of visits received at least one analgesic (opioid or non-opioid) treatment. 33.4% of visits for suspected appendicitis received an opioid.

Table 2 shows the distribution of analgesia receipt by race/ethnicity. In bivariate analyses (Appendix 4), Black NH children were more likely to receive any analgesic (OR = 1.28 (95% CI, 1.04–1.56)] and only non-opioid analgesia [OR = 1.58 (95% CI, 1.28–1.94)] than White NH children. Hispanic children were more likely to receive any analgesic treatment [OR = 1.43 (95% CI, 1.22–1.68)] and only non-opioid analgesia than White NH children [OR = 1.59 (95% CI, 1.35–1.88)]. The “other” racial group children were less likely to receive an opioid analgesia than White NH children [OR = 0.63 (95% CI, 0.46–0.85)]. The utilization of pain medications by initial pain score and by race/ethnicity are shown in Appendix 2.

#### Analgesic Treatment and Opioid Analgesia: Multivariate Analyses

In the multivariate analyses (Table 5), there were no significant differences in the likelihood to receive any analgesic treatment between Black NH and White NH children [aOR = 1.09 (95%CI, 0.86–1.38)] but Black NH children were less likely to receive an opioid analgesia than White NH children [aOR = 0.75 (95% CI, 0.58–0.96)]. There were no significant differences in the likelihood to receive any analgesic treatment between Hispanic and White NH children [aOR = 1.21 (95% CI, 0.99–1.47)] but Hispanic children were less likely to receive an opioid analgesia than White NH children [aOR = 0.78 (95% CI, 0.63–0.96)]. Children in the “other” racial group were less likely to receive an opioid analgesia than White NH children [aOR = 0.70 (95% CI, 0.50–0.99)].

**Table 5:** Factors associated with the administration of any analgesic treatment and opioid analgesia for children visiting the emergency department with suspected appendicitis.

	**Any analgesic treatment**	**Opioid analgesia**
	**OR (95%-CI)**	**OR (95%-CI)**
**Race, ethnicity %**		
White non-Hispanic	1	1
Black non-Hispanic	1.09 (0.86–1.38)	**0.75 (0.58–0.96)**
Hispanic	1.21 (0.99–1.47)	**0.78 (0.63–0.96)**
Other	0.89 (0.66–1.19)	**0.70 (0.50–0.99)**
**Sex**		
Male	1	1
Female	**1.24 (1.08–1.42)**	0.97 (0.84–1.13)
**Age, years**	1.00 (0.98–1.02)	**1.07 (1.05–1.09)**
**Insurance status**		
Public	1	1
Private	1.01 (0.86–1.19)	0.90 (0.76–1.07)
No insurance	2.38 (0.94–6.01)	1.88 (0.85–4.17)
**Median household income by ZIP code in $, mean**	1.00 (1.00–1.00)	1.00 (1.00–1.00)
**Triage score**		
1–2	1	1
3	**0.59 (0.46–0.75)**	**0.53 (0.42–0.66)**
4–5	**0.56 (0.35–0.88)**	**0.19 (0.10–0.36)**
**Pain score group, %**		
None (0)	1	1
Mild (1–3)	1.16 (0.89–1.50)	1.14 (0.80–1.61)
Moderate (4–6)	**1.87 (1.50–2.31)**	**1.91 (1.43–2.55)**
Severe (7–10)	**3.54 (2.82–4.44)**	**4.48 (3.36–5.98)**
**Surgery for appendicitis**		
No	**1**	**1**
Yes	1.15 (0.99–1.33)	**3.06 (2.62–3.59)**

*Multivariate analyses. Bold values indicate statistically significant*.

## Discussion

The present study shows racial/ethnic disparities in pain management across two different painful conditions. Specifically, our findings show that Black NH children and Hispanic children are more likely to present with severe initial pain but were less likely to receive an opioid analgesia than White NH children. The consistency of these findings across two different conditions within a single institution are concerning for healthcare inequities rooted in racism.

There are fewer studies investigating analgesic racial/ethnic disparities in children visiting the ED than for adults. Most of these studies either used data from the National Hospital Ambulatory Medical Care Survey (NHAMCS), an annual nationwide survey designed to collect ED service data (4–8), or involved single center studies with small sample size (10–13). Prior work looking at disparities in pain management of patients with long bone fractures shows heterogeneous results. Whereas one study using NHAMCS data from 1992 to 1998 (8) and two single center studies (11, 12) found no differences in opioid administration between White NH and children of color, one recent multicenter study found that children of color were less likely to receive an opioid treatment when visiting the PED with a long bone fracture (9). The same heterogeneous results are observed regarding disparities in pain management of abdominal pain and appendicitis. From the NHAMCS data, Black children were found to receive less opioid treatment than White children during visits for abdominal pain from 2006 to 2009 (15) and for appendicitis from 2003 to 2010 (6), whereas others found no racial differences in opioid administration in patients 12–55 years old during a visit for appendicitis or gallbladder disease between 2010 and 2014 (7). Caperell et al. included 9,424 children with abdominal pain visiting one PED and observed no significant racial differences in analgesia when studying specifically children with a diagnosis of appendicitis (*n* = 404 with only 36 Black NH children) (10).

We included in our study two large cohorts of patients with enough power to test a racial/ethnic difference of opioid administration with at least an OR of 0.8, similar to the magnitude found in studies using the NHAMCS (4–6, 15). We found differences in opioid administration for Black NH and Hispanic children compared to White NH children for both conditions, which suggest the existence of actual racial/ethnical inequities in opioid administration independent of the type of painful condition.

In contrast to the lower use of opioids, we found that Black NH children were more likely to receive any analgesic treatment than White NH children in our fracture cohort and Hispanic children were more likely to have any analgesic treatment than White NH children in the suspected appendicitis cohort. Goyal et al. also found in a recent study that minority children were more likely to receive an analgesic treatment in visits for long-bone fracture (9). We were not able to gather the information whether children took an analgesic treatment before coming to the ED. The fact that White NH children are more likely to take an analgesic treatment before coming to the ED (25) might explain this difference, however given the duration of time of the average ED visit for the work up of appendicitis or fracture management, we would expect these children to ultimately receive non-opioid analgesics during their visit.

Black NH children and Hispanic children were more likely to receive a non-opioid analgesia than White NH children, mainly for moderate and severe pain. This suggests that although pain is recognized, physicians react differently by treating Black NH and Hispanic children with non-opioid treatment while treating White NH children with opioid analgesia for similar pain levels and etiologies of pain. Goyal et al. found the same pattern between Black and White children visiting the ED with appendicitis (34.1 vs. 13.9% for non-opioid analgesia and 20.7 vs. 43.1% for opioid analgesia, respectively, for Black and White children) (6). Liao and Reyes showed also that Black children with long bone fracture were more likely to receive a non-opioid treatment than White children though the difference was not statistically significant (39 vs. 32%, respectively) (12).

Disparities has been defined by the Institute of Medicine as “observed differences in quality of healthcare by race/ethnicity that are not due to access to clinical needs or appropriateness of the intervention” (26). Health inequities, on the other hand, are differences in health or medical treatment that are not only unnecessary and avoidable but, in addition, are considered unfair and unjust. Health inequities are rooted in social injustices that make some population groups more vulnerable to poor health than other groups (27, 28). So racial/ethnic differences in opioid use may reflect clinical appropriateness, patient preferences, or represent actual inequities *via* over-prescription of opioids to White NH children, or under-treatment of Black and Hispanic children (29). Regarding patients preferences, while the presence of a high pain score may not translate to the desire for a given analgesic treatment (30), no differences by race/ethnicity in analgesia desire in the ED has been demonstrated in adults (31). However, some studies suggest that race and ethnic differences may exist for preference of type of analgesia (32). For example, Hispanic patients may have greater reluctance to use opioids than White NH patients (33). However, to the best of our knowledge, no studies have investigated patients/parents' analgesia desire and type of analgesia preferences for acute pain in children in an ED setting.

It is more plausible that those differences reflect at least in part an actual racial/ethnic inequity in pain management. This has been demonstrated for other interventions which show that White NH children were more likely to receive unnecessary medical assessment and interventions (34–36). For example, Goyal et al. demonstrated that White NH children were more likely to receive unnecessary antibiotics than Black NH and Hispanic children for viral acute respiratory tract infection (34). However, opioid treatment remains a cornerstone in the management of patient with moderate or severe pain for both long bone fractures and appendicitis (37). The differences in our study most likely represent true racial inequities with an under prescription of opioids for Black NH and Hispanic children.

Racial and ethnic disparities in the PED have also been demonstrated in other conditions. For example, non-White adolescents are more likely to be tested for sexually transmitted infections than White adolescents (35) and physicians are more likely to order laboratory and radiologic tests for White children in comparison to non-White children (38). White children have higher triage acuity scores than their Black counterparts for the same conditions, have shorter wait times and are less likely to leave the emergency department without being seen (39–41). Why these disparities exist is not clear. The nature of work in an ED can pose limitations to the delivery of high quality and equitable care because of the time pressure and high cognitive load required. Physicians must make time-sensitive decisions and manage multiple patients simultaneously. These environmental characteristics may make physicians more prone to decisions that are subconsciously influenced by racial and ethnic biases (42–46).

Causes of disparities and ultimately inequities are complex and multifactorial. Provider's implicit bias—unconscious negative attitudes and stereotypes toward specific groups—has been identified as potentially implicated in disparities of care (47–49). Studies utilizing the Implicit Association Test (IAT), a widely used instrument to assess implicit bias (50), demonstrate that most physicians, including pediatricians, display a pro-White bias (vs. Black and Hispanic) for adults and children (51–53). However, the impact of implicit bias on clinical decision making is still unclear and has primarily been studied using clinical vignette studies, not real-life patient management. Further studies should evaluate the role of implicit bias in the management of actual patients.

Our study has several limitations. First, the selection of the fracture cohort *via* discharge diagnoses codes may not be 100% sensitive nor specific. However, there is no reason to assume any inaccuracy in cohort selection would preferentially affect individuals of a specific race or ethnicity. Second, because the study is retrospective, we have no information regarding the reasons for pain medication choices, which can be related to patient preference, physician preference, explicit or implicit bias of the physicians or nurses, the relative activity of ED or other unmeasured/unknown factors. We were unable to investigate whether some patients declined an opioid treatment and whether that differed between patients of different races/ethnicities. The “third wave” of the opioid epidemic has been noted to start around 2013, which may have affected patient and provider preferences. However, these differences in pain management came about, they still represent widespread and consistent inequities in the medical management of pain between children of different races/ethnicities. Third, regarding the outcome of pain management, we investigated the analgesia modalities during the whole visit but adjusted only for the initial pain score and were unable to assess the evolution of the pain during the visit. Fourth, patients that were transferred to the emergency department from outside hospitals may have received analgesia prior to arrival. However, a pain score would still be assessed upon arrival in the ED and pain management decisions would be made using that assessment. Though we were broadly inclusive in analytic inclusion of confounding factors we cannot exclude some residual confounding. Our data included visits from 2011 to 2015, and the differences we found may have changed over time. Finally, even though our results are consistent with findings from national studies, given the single institution nature of our study the results may not be generalizable to other EDs especially general EDs as opposed to PEDs.

In conclusion, we found that Black NH children and Hispanic children were less likely to receive an opioid analgesia when visiting the ED for a painful condition than White NH children, and at the same time were more likely to receive a non-opioid analgesia when presented with moderate or severe pain. Our data bring a new piece of evidence of racial/ethnic inequities that may reflect the effects of racial discrimination in the analgesic management of children in the ED.

## Prior Presentation

The results from this study were presented at the Pediatric Academic Societies Annual Meeting 2017, in San Francisco.

## Data Availability Statement

The raw data supporting the conclusions of this article will be made available by the authors, without undue reservation.

## Ethics Statement

The studies involving human participants were reviewed and approved by Boston Children's Hospital Institutional Review Board. Written informed consent from the participants' legal guardian/next of kin was not required to participate in this study in accordance with the national legislation and the institutional requirements.

## Author Contributions

RG, JK, CB, AK, and EF conceptualized and designed the study. RG and AK coordinated and supervised the data collection. RG performed the statistical analyses, drafted the initial manuscript, and takes responsibility for the paper as a whole. JK, CB, AK, EF, and MM contributed substantially to its revision. All authors contributed to the article and approved the submitted version.

## Conflict of Interest

The authors declare that the research was conducted in the absence of any commercial or financial relationships that could be construed as a potential conflict of interest.

## Publisher's Note

All claims expressed in this article are solely those of the authors and do not necessarily represent those of their affiliated organizations, or those of the publisher, the editors and the reviewers. Any product that may be evaluated in this article, or claim that may be made by its manufacturer, is not guaranteed or endorsed by the publisher.
