# Supplementary material for: Racial and Ethnic Disparities in Pain Management of Children With Limb Fractures or Suspected Appendicitis: A Retrospective Cross-Sectional Study
Source: Front Pediatr. 2021 Aug 3;9:652854. doi: 10.3389/fped.2021.652854 (PMC8369476; doi:10.3389/fped.2021.652854)
Supplement: Supplementary file 1 [file Presentation_1.PPTX]

## Slide 1
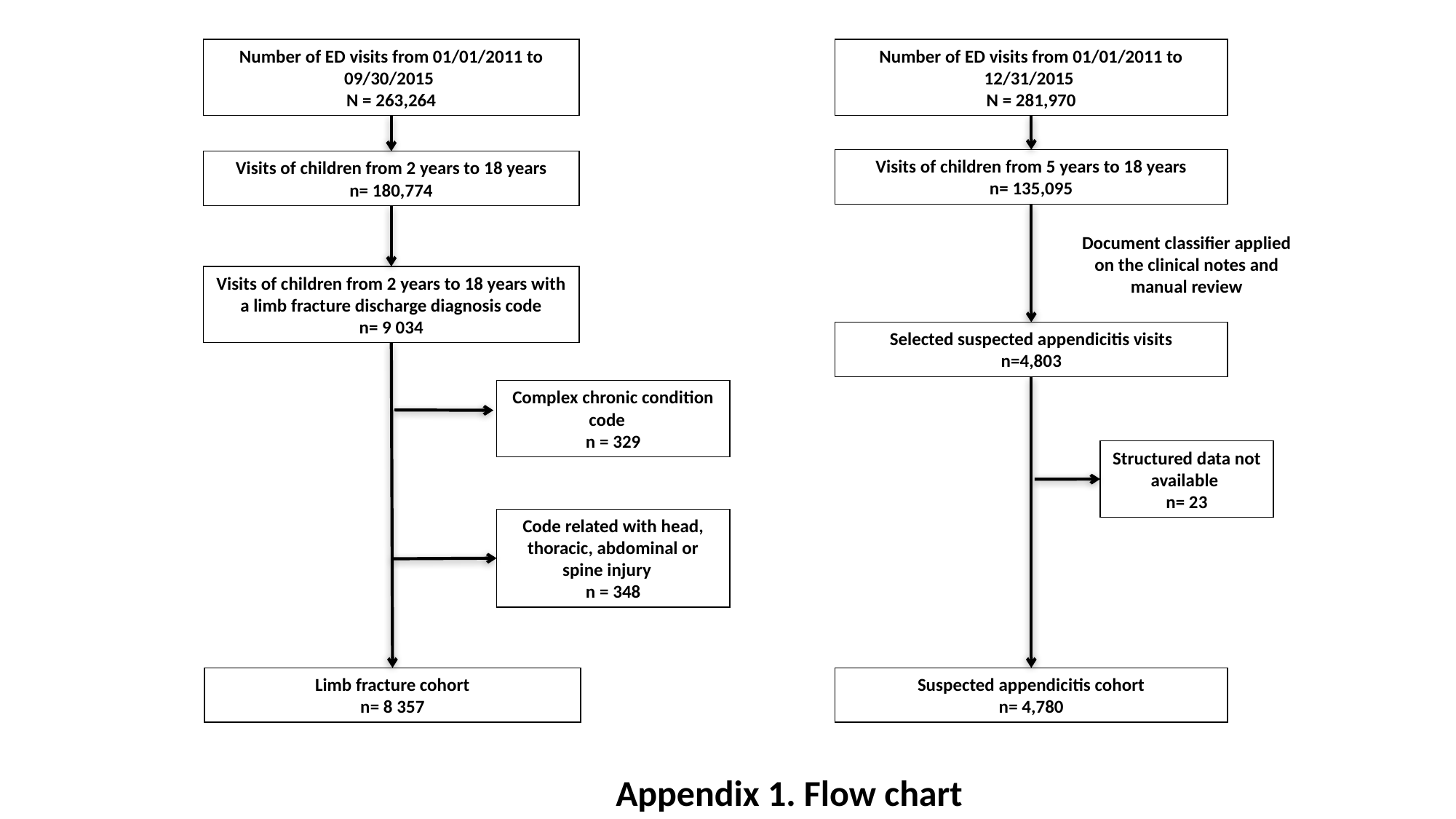

Number of ED visits from 01/01/2011 to 09/30/2015
N = 263,264
Number of ED visits from 01/01/2011 to 12/31/2015
N = 281,970
Visits of children from 5 years to 18 years
n= 135,095
Visits of children from 2 years to 18 years
n= 180,774
Document classifier applied on the clinical notes and manual review
Visits of children from 2 years to 18 years with a limb fracture discharge diagnosis code
n= 9 034
Selected suspected appendicitis visits
n=4,803
Complex chronic condition code
n = 329
Structured data not
available
n= 23
Code related with head, thoracic, abdominal or spine injury
n = 348
Suspected appendicitis cohort
n= 4,780
Limb fracture cohort
n= 8 357
Appendix 1. Flow chart
