# Supplementary material for: Racial and Ethnic Disparities in Pain Management of Children With Limb Fractures or Suspected Appendicitis: A Retrospective Cross-Sectional Study
Source: Front Pediatr. 2021 Aug 3;9:652854. doi: 10.3389/fped.2021.652854 (PMC8369476; doi:10.3389/fped.2021.652854)
Supplement: Supplementary file 2 [file Presentation_2.PPTX]

## Slide 1
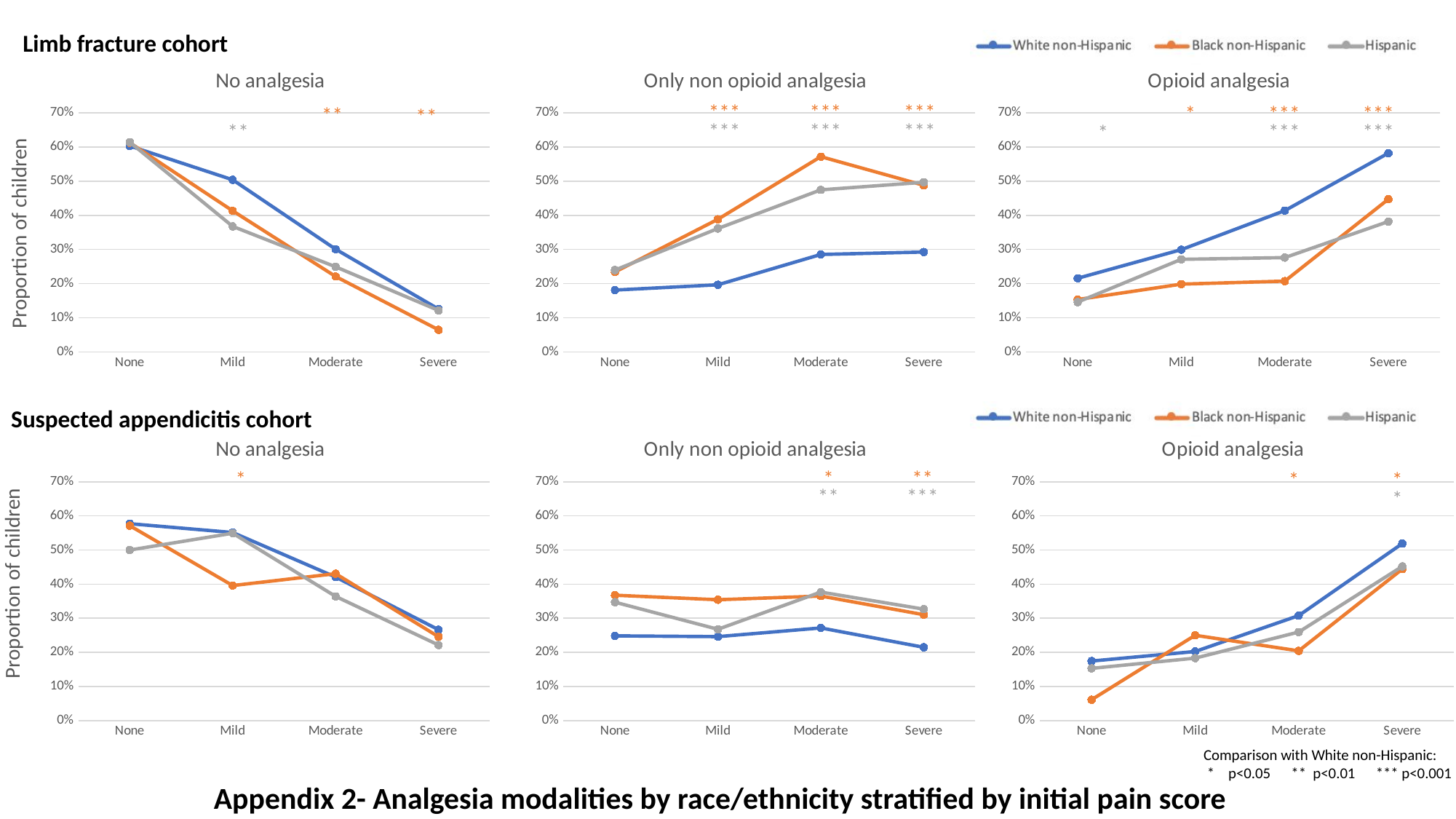

Limb fracture cohort
### Chart: No analgesia
| Category | No analgesia | No analgesia | No analgesia |
|---|---|---|---|
| None | 0.603363 | 0.6129032 | 0.614583 |
| Mild | 0.5041322 | 0.4132231 | 0.3674699 |
| Moderate | 0.3007743 | 0.2207207 | 0.2491582 |
| Severe | 0.1252144 | 0.0644068 | 0.1212121 |
### Chart: Only non opioid analgesia
| Category | Non opioid | Non opioid | Non opioid |
|---|---|---|---|
| None | 0.181008 | 0.233871 | 0.2395833 |
| Mild | 0.1965106 | 0.3884298 | 0.3614458 |
| Moderate | 0.2852889 | 0.5720721 | 0.4747475 |
| Severe | 0.2924528 | 0.4881356 | 0.4969697 |
### Chart: Opioid analgesia
| Category | Opioid | Opioid | Opioid |
|---|---|---|---|
| None | 0.2156281 | 0.1532258 | 0.1458333 |
| Mild | 0.2993572 | 0.1983471 | 0.2710843 |
| Moderate | 0.4139369 | 0.2072072 | 0.2760943 |
| Severe | 0.5823328 | 0.4474576 | 0.3818182 |Proportion of children
***
***
***
***
*
***
**
**
***
***
***
***
**
***
*
Suspected appendicitis cohort
### Chart: No analgesia
| Category | No analgesia | No analgesia | No analgesia |
|---|---|---|---|
| None | 0.5771812 | 0.5714286 | 0.5 |
| Mild | 0.5514019 | 0.3958333 | 0.5492958 |
| Moderate | 0.4208417 | 0.4306569 | 0.3640167 |
| Severe | 0.2662857 | 0.2456897 | 0.2214765 |
### Chart: Only non opioid analgesia
| Category | Non opioid | Non opioid | Non opioid |
|---|---|---|---|
| None | 0.2483221 | 0.3673469 | 0.3469388 |
| Mild | 0.2461059 | 0.3541667 | 0.2676056 |
| Moderate | 0.2715431 | 0.3649635 | 0.376569 |
| Severe | 0.2148571 | 0.3103448 | 0.3266219 |
### Chart: Opioid analgesia
| Category | Opioid | Opioid | Opioid |
|---|---|---|---|
| None | 0.1744966 | 0.0612245 | 0.1530612 |
| Mild | 0.2024922 | 0.25 | 0.1830986 |
| Moderate | 0.3076152 | 0.2043796 | 0.2594142 |
| Severe | 0.5188571 | 0.4439655 | 0.4519016 |Proportion of children
**
*
*
*
*
**
***
*
Comparison with White non-Hispanic:
 * p<0.05 ** p<0.01 *** p<0.001
Appendix 2- Analgesia modalities by race/ethnicity stratified by initial pain score
