# Supplementary material for: Racial and Ethnic Disparities in Pain Management of Children With Limb Fractures or Suspected Appendicitis: A Retrospective Cross-Sectional Study
Source: Front Pediatr. 2021 Aug 3;9:652854. doi: 10.3389/fped.2021.652854 (PMC8369476; doi:10.3389/fped.2021.652854)
Supplement: Supplementary file 3 [file Table_1.DOCX]

Appendix 3- Factors associated with the administration of any analgesic treatment (opioid and non-opioid) and opioid analgesia for children visiting the emergency department with a limb fracture. Bivariate analyses

|  | Any analgesic treatment  n/N % | P* | Opioid analgesia  n/N % | P* |
| --- | --- | --- | --- | --- |
| **Total** | 5,431/8,357 65.0% |  | 3,001 / 8,357 35.9% |  |
| **Race, ethnicity %**  White non-Hispanic  Black non-Hispanic  Hispanic  Other | 3,151/4,989 63.2%  574/ 772 74.4%  697/ 996 70.0%  288/ 450 64.0% | **<0.001** | 1,927/4,989 38.6%  221/ 772 28.6%  282/ 996 28.3%  142/ 450 31.6% | **<0.001** |
| **Sex**  Male  Female | 3,267/5,001 65.3%  2,164/3,356 64.5% | 0.4 | 1,913/5,001 38.3%  1,088/3,356 32.4% | **<0.001** |
| **Age, years** |  | **<0.001** |  | **<0.001** |
| **Insurance status**  Public  Private  No insurance | 1,649/2,542 64.9%  3,739/5,756 65.0%  43/ 59 73.0% | 0.4 | 825/2,542 32.5%  2,153/5,756 37.4%  23/ 59 39.0% | **<0.001** |
| **Median household income by ZIP code in $, mean** |  | **<0.05** |  | **<0.01** |
| **Triage score**  1-2  3  4-5 | 570/ 695 82.0%  4,451/6,893 64.6%  388/ 709 54.7% | **<0.001** | 518/ 695 74.5%  2,426/6,893 35.2%  40/ 709 5.6% | **<0.001** |
| **Pain score group, %**  None  Mild  Moderate  Severe | 660/1,686 39.2%  904/1,727 52.4%  1,949/2,726 71.5%  1,885/2,135 88.3% | **<0.001** | 334/1,686 19.8%  506/1,727 29.3%  1,027/2,726 37.7%  1,120/2,135 52.5% | **<0.001** |
| **Fracture Location**  Lower limb  Upper limb | 1,521/2,206 69.0%  3,910/6,151 63.6% | **<0.001** | 894/2,206 40.5%  2,107/6,151 34.3% | **<0.001** |
| **Sedation**  No  Yes | 4,194/6,511 64.4%  1,237/1,846 67.0% | **<0.05** | 2,017/6,511 31.0%  984/1,846 53.3% | **<0.001** |

* p value for bivariate analyses. Chi^2^ test for categorial variable and t test for continuous variable
