# Supplementary material for: Racial and Ethnic Disparities in Pain Management of Children With Limb Fractures or Suspected Appendicitis: A Retrospective Cross-Sectional Study
Source: Front Pediatr. 2021 Aug 3;9:652854. doi: 10.3389/fped.2021.652854 (PMC8369476; doi:10.3389/fped.2021.652854)
Supplement: Supplementary file 4 [file Table_2.DOCX]

Appendix 4- Factors associated with the administration of any analgesic treatment (opioid and non-opioid) and opioid analgesia for children visiting the emergency department with a suspected appendicitis. Bivariate analyses

|  | Any analgesic treatment  n/N % | P* | Opioid analgesia  n/N % | P* |
| --- | --- | --- | --- | --- |
| **Total** | 2,910/4,780 60.9% |  | 1,598/4,780 33.4% |  |
| **Race, ethnicity %**  White non-Hispanic  Black non-Hispanic  Hispanic  Other | 1,541/2,599 59.3%  316/ 486 65.0%  608/ 900 67.6%  129/ 235 54.9% | **<0.001** | 906/2,599 34.9%  152/ 486 31.3%  302/ 900 33.6%  59/ 235 25.1% | **<0.05** |
| **Sex**  Male  Female | 1,178/2,077 56.7%  1,732/2,703 64.1% | **<0.001** | 692/2,077 33.3%  906/2,703 33.5% | 0.9 |
| **Age, years** |  | **<0.001** |  | **<0.001** |
| **Insurance status**  Public  Private  No insurance | 1,006/1,603 62.8%  1,872/3,134 59.7%  26/ 32 81.3% | **<0.01** | 535/1,603 33.4%  1,044/3,134 33.3%  15/ 32 46.9% | 0.3 |
| **Median household income by ZIP code in $, mean** |  | **<0.001** |  | 0.5 |
| **Triage score**  1-2  3  4-5 | 339/ 454 74.7%  2,483/4,170 59.5%  76/ 134 56.7% | **<0.001** | 234/ 454 51.5%  1,340/4,170 32.1%  16/ 134 11.9% | **<0.001** |
| **Pain score group, %**  None  Mild  Moderate  Severe | 240/ 546 44.0%  255/ 553 46.1%  969/1,682 57.6%  1,339/1,800 74.4% | **<0.001** | 86/ 546 15.8%  109/ 553 19.7%  475/1,682 28.2%  877/1,800 48.7% | **<0.001** |
| **Surgery for appendicitis**  No  Yes | 2,040/3,369 60.6%  870/1,411 61.7% | 0.5 | 915/3,369 27.2%  683/1,411 48.4% | **<0.001** |

* p value for bivariate analyses. Chi^2^ test for categorial variable and t test for continuous variable
